# Combined Genomic and Genetic Data Integration of Major Agronomical Traits in Bread Wheat (*Triticum aestivum* L.)

**DOI:** 10.3389/fpls.2017.01843

**Published:** 2017-11-14

**Authors:** Umar M. Quraishi, Caroline Pont, Qurat-ul Ain, Raphael Flores, Laura Burlot, Michael Alaux, Hadi Quesneville, Jerome Salse

**Affiliations:** ^1^Department of Plant Sciences, Quaid-i-Azam University, Islamabad, Pakistan; ^2^Institut National de la Recherche Agronomique, Université Clermont Auvergne, UMR 1095 Génétique, Diversité et Ecophysiologie des Céréales, Clermont-Ferrand, France; ^3^Institut National de la Recherche Agronomique UR1164 URGI (Research Unit in Genomics-Info), Université Paris-Saclay, Versailles, France

**Keywords:** wheat, traits, gene, QTL, synteny

## Abstract

The high resolution integration of bread wheat genetic and genomic resources accumulated during the last decades offers the opportunity to unveil candidate genes driving major agronomical traits to an unprecedented scale. We combined 27 public quantitative genetic studies and four genetic maps to deliver an exhaustive consensus map consisting of 140,315 molecular markers hosting 221, 73, and 82 Quantitative Trait Loci (QTL) for respectively yield, baking quality, and grain protein content (GPC) related traits. Projection of the consensus genetic map and associated QTLs onto the wheat syntenome made of 99,386 genes ordered on the 21 chromosomes delivered a complete and non-redundant repertoire of 18, 8, 6 metaQTLs for respectively yield, baking quality and GPC, altogether associated to 15,772 genes (delivering 28,630 SNP-based makers) including 37 major candidates. Overall, this study illustrates a translational research approach in transferring information gained from grass relatives to dissect the genomic regions hosting major loci governing key agronomical traits in bread wheat, their flanking markers and associated candidate genes to be now considered as a key resource for breeding programs.

## Introduction

The development of high-yielding, durably stress-tolerant wheat varieties is essential to ensure present and future food security in coping to ongoing and future climate change (Boyer and Westgate, 2004; Heijmans et al., 2005; Habash et al., 2009). This can only be achieved through the identification of the genetic bases of key traits and their proper utilization in genomics-assisted breeding programs. Bread wheat (*Triticum aestivum* L.), 3rd cereal for production and 1st for world trade, has been a central crop for the development of numerous genetic and genomic resources by the scientific community during the last decades to reach this objective (Borrill et al., 2015). However, very few genetic/genomic information have been effectively transferred into breeding programs due to the lack of an integrative framework of the existing resources, references to as meta-analysis.

Meta-analysis is the statistical concept of integrating in a single analysis different data obtained independently. The meta-analysis concept was transposed by Goffinet and Gerber (2000) into the field of genetics and especially into the concept of the calculation of meta- Quantitative Trait Loci (QTL) from independent studies (Veyrieras et al., 2007). The method allows the length of the confidence interval of QTL location to be consistently reduced when there is co-localization of several QTL loci deriving from independent quantitative genetic studies. This derived method (meta-QTL, hereafter MQTL) was successfully carried out on dairy cattle (MacLeod et al., 2003; Khatkar et al., 2004; Charbonneau et al., 2006; Lean et al., 2006), Human (Heijmans et al., 2005; Lawlor et al., 2006; Rice et al., 2006), as well as in the field of plant breeding and more precisely for the genetic determinisms of flowering time in maize (Chardon et al., 2004, 2005; Salvi et al., 2011) and wheat (Hanocq et al., 2007; Griffiths et al., 2009; Gegas et al., 2010; Tyagi et al., 2015).

Genetic resources and associated genome-wide diversity maps have been made publicly available for hexaploids (Chao et al., 2010; Allen et al., 2011, 2013; Lai et al., 2012; Winfield et al., 2012; Cavanagh et al., 2013), tetraploids (Saintenac et al., 2011; Trebbi et al., 2011), or diploid (You et al., 2011; Wang et al., 2013) wheats. Despite the previous genetic data accumulated during the last decade, wheat genomic resources have also been recently published with the release of the genome shotgun sequences of hexaploid (Brenchley et al., 2012; International Wheat Genome Sequencing Consortium, 2014) and diploid (Jia et al., 2013; Ling et al., 2013; Luo et al., 2013) wheats. The access to such public resources offers now the opportunity to unveil, to an unprecedented scale, the molecular mechanisms driving major agronomical traits in wheat in conducting a meta-analysis integrating genetic (markers and QTLs) and genomic (gene and genome sequences) data to deliver a completed catalog of markers and putative candidate genes driving such traits.

A large bibliographic survey allowed us to identify more than 90 publications for agronomic traits in bread wheat (excluding biotic and abiotic stresses). Out of these, 27 (~30%) publications provided the necessary information for MQTL calculation such as, the genetic mapping (marker name, position) as well as QTL information (LOD score, confidence interval, position on the genetic map, R^2^) for grain yield (with grain number, tons per hectare, tiller number, thousand kernel weight, harvest index, plant height), grain protein content (GPC), and baking quality (with pentosan viscosity, dough strength, volume, panification, durity, height of bubble in alveograph, pel-schank, total starch, flour swelling volume, particule size index, slope after peak of mixograph) traits (Charmet et al., 2001; Börner et al., 2002; Groos et al., 2003, 2007; Prasad et al., 2003; Huang et al., 2004, 2006; Turner et al., 2004; Kulwal et al., 2005; McCartney et al., 2005, 2006; Kumar et al., 2006; Narasimhamoorthy et al., 2006; Laperche et al., 2007; Li et al., 2007; Chu et al., 2008; Cuthbert et al., 2008; Sun et al., 2008; Wang et al., 2009, 2011; Deng et al., 2011; Tang et al., 2011; Bennett et al., 2012; Mir et al., 2012; Liu et al., 2013). From these genetic and genomic resources, we deliver in the current study (i) the construction of a dense composite genetic map on which major public genetic maps has been integrated; (ii) the projection of public QTLs for yield, baking quality and GPC from different populations; (iii) the calculation of consensus MQTLs; (iv) the identification of candidate genes exploiting the synteny with grass relatives.

## Results

### High resolution wheat consensus genetic map

**Figure 1:**
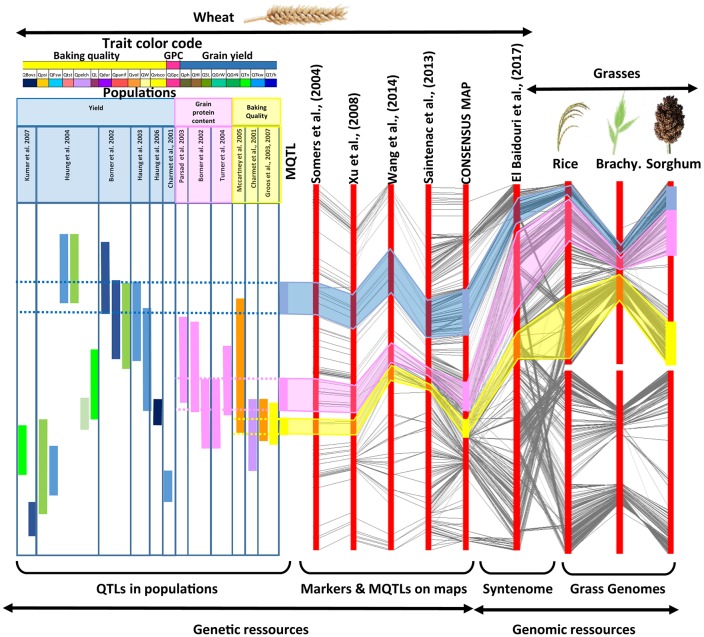
Strategy for genomic and genetic resources integration. The figure, from left to right, illustrates the integration of (i) independent QTL analyses from distinct mapping populations deriving MQTLs (with a color code for the considered traits and populations, top), (ii) genetic maps deriving a consensus high density map (with conserved markers linked with black connecting lines), (iii) the syntenome deriving from the exploitation of the synteny with relatives (with rice, *Brachypodium* and sorghum orthologs linked with black connecting lines). This strategy fills the gap between low resolution QTL intervals in a species of interest (left) and known gene functions in closely related species for a considered trait (right).

**Figure 2:**
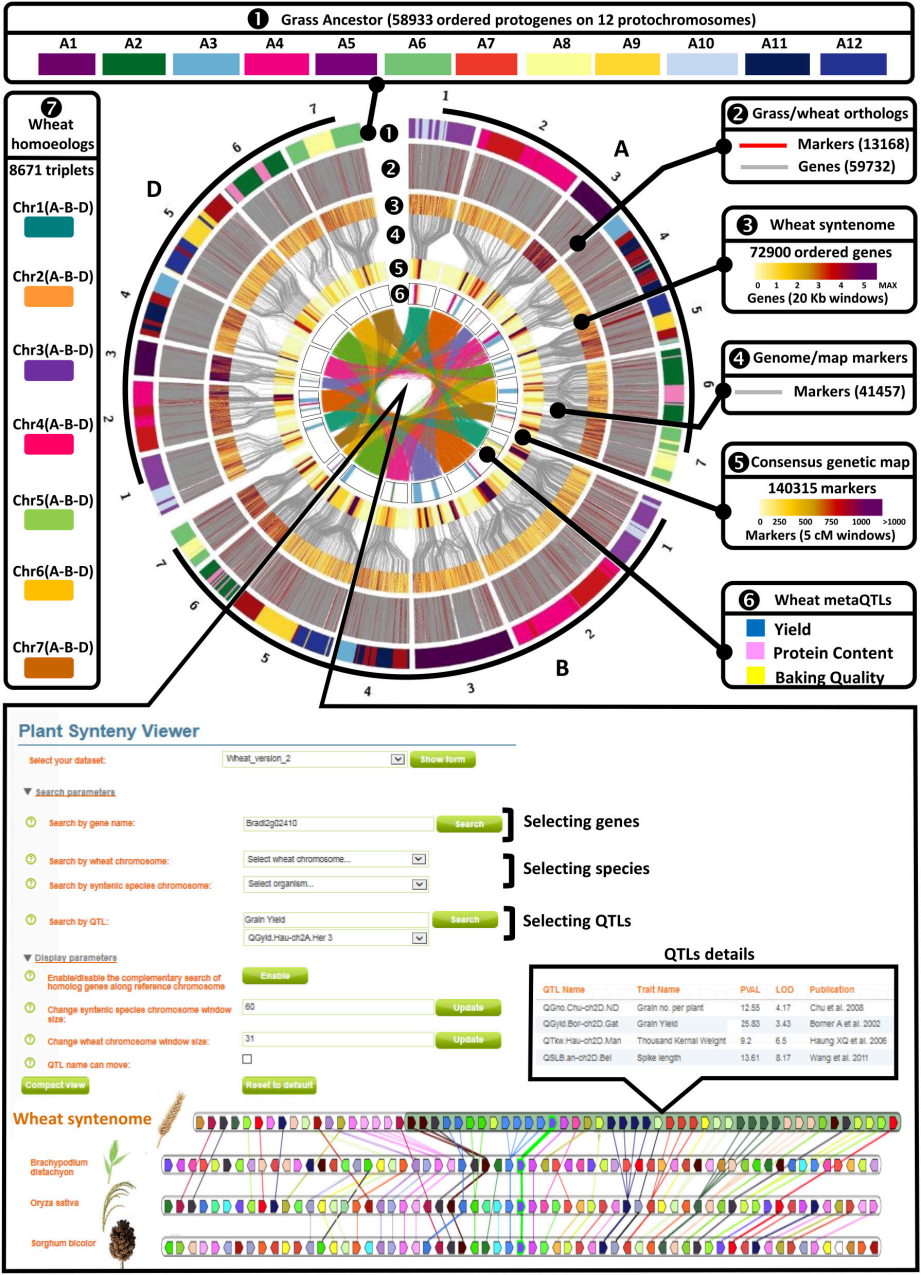
Genomic and genetic integration of major agronomical traits in bread wheat. A—*Wheat genomic and genetic resources. Circle 1*—Illustration of the synteny between the *n* = 12 AGK (color code for A1–A12) and the 21 bread wheat chromosomes (1–21). *Circle 2*—Illustration of the wheat genes ordered on the 21 chromosomes based on molecular markers (red connecting lines) and synteny with AGK (gray connecting lines). *Circle 3*—Heat map illustration of the gene density (color code in legend for the number of genes within 20 kbp physical windows) on the 21 chromosomes. *Circle 4*—Molecular markers bridging (gray connecting lines) the consensus genetic map to the syntenome. *Circle 5*—Heat map illustration of the marker density (color code in legend for the number of markers within 5 cM genetic intervals) on the 21 chromosomes. *Circle 6*—Illustration of the MQTL intervals with a color code for yield (blue), protein content (pink), and baking quality (yellow). *Center* 7—Illustration of the retained homoeologous triplets (A, B, and D copies) on the 21 chromosomes. B—*Wheat web viewer*. Screen capture of the PlantSyntenyViewer web tool [http://urgi.versailles.inra.fr/synteny-wheat] visualizing the synteny between wheat, *Brachypodium*, rice, sorghum and delivering the access to the wheat syntenome made of 72,900 genes ordered on the 21 chromosomes as well as the information (populations, traits, flanking markers, trial details) related to the 376 QTLs, 32 MQTLs, and 37 candidate genes described in the current study.

In order to integrate public quantitative genetic studies of traits (Figure 1), we constructed a high resolution consensus genetic map using Biomercator v3.0 software (Sosnowski et al., 2012), removing markers showing inconsistency in their positions between the considered genetic maps, due to errors in mapping or genotype-specific inversions and translocations, using mapinspect v2.0 software. Following this strategy, four public genetic maps have been integrated consisting in 2,293 markers from Xu et al. (2008), 1,239 markers from Somers et al. (2004), 40,267 markers from Wang et al. (2014) and 104,804 markers from Saintenac et al. (2013). The derived consensus high-resolution genetic map is made of 140,315 molecular markers (with an average of 6,682 markers per chromosome) and a recombination distance of 4,853.22 cM (1,687.22, 1,489.19, and 1,676.80 cM for the A, B, and D subgenomes respectively), Figure 2 and Table 1. The highest number of markers were obtained on the homoeologous group 2 (23,311) and the B subgenome (55,524) with the lowest number of markers found on the homoeologous group 4 (14,175) and the D subgenome (38,058). The consensus genetic map, consisting in SSRs (4,367), RFLPs (2,001), DarTs (2,231), Genes (284) and SNPs (131,432), is made available as Supplementary Table 1.

**Table 1:** Wheat consensus genetic map.

**Chr**	**SNP**	**SSR**	**RFLP**	**DarT**	**Gene**	**Total**
1A	6,002	268	112	121	23	6,526
1B	8,336	311	139	169	31	8,986
1D	3,795	193	94	57	16	4,155
2A	6,148	224	129	75	16	6,592
2B	8,837	223	119	163	24	9,366
2D	6,950	209	110	68	16	7,353
3A	6,284	199	105	97	8	6,693
3B	8,115	333	114	387	17	8,966
3D	6,585	154	81	45	7	6,872
4A	5,597	226	104	113	11	6,051
4B	4,566	136	63	46	7	4,818
4D	3,143	86	61	15	1	3,306
5A	7,012	182	91	54	9	7,348
5B	8,077	260	100	135	12	8,584
5D	5,996	142	53	26	9	6,226
6A	5,242	187	86	87	7	5,609
6B	6,258	226	107	135	8	6,734
6D	4,120	128	72	28	5	4,353
7A	7,336	263	109	183	22	7,913
7B	7,496	277	106	179	13	8,071
7D	5,537	140	46	48	22	5,793
Total	13,1432	4,367	2,001	2,231	284	140,315

*The table delivers the detailed information (i.e., chromosomes in rows and marker types in columns) for the 140,315 makers of the wheat consensus genetic map*.

### Major MetaQTLs of wheat traits

Twenty-seven independent quantitative genetic studies delivering 376 QTLs related to grain yield (GY), grain protein content (GPC) and baking quality (BQ) were projected onto the previous high resolution consensus genetic map (Figure 1 and Table 2). Two hundred and twenty-one QTLs referenced to as GY are associated to yield components such as grain weight (GrW), grain number (GrN), tons per hectare (T/h), tiller number (Tn), thousand kernel weight (TKW), harvest index (HI), plant height (PH), spike length (SL). Seventy three QTLs referenced to as BQ are associated to baking quality related traits such as pentosan viscosity (Visco.), dough strength (W), volume (Vol.) as well as other traits such as (panification, durity, height of bubble in alveograph, pel-schank, total starch, flour swelling volume, particule size index, slope after peak of mixograph). Finally, 82 QTLs referenced to as GPC are related to grain protein content and composition and were integrated on the consensus genetic map.

**Table 2:** Wheat populations and QTLs.

**Populations**					**Yield**								**Quality**				**GPC**
**P1**	**P2**	**Size**	**Type**	**Lines**	**T/h**	**TKW**	**Tn**	**GrN**	**HI**	**PH**	**SL**	**GrW**	**Visco**	**W**	**Vol**	**Other**	
Apache	Ornicar	222	DH	176	12	–	–	–	–	–	–	–	–	–	–	–	20
Courtot	chinese spring	187	DH	662	–	7	–	–	–	–	–	–	8	5	–	10	–
Opata 85	W7984	114	RILs	511	–	11	–	10	–	–	–	17	—	–		–	2
Opata 85	W7984	110	RILs	358	–	–	–	–	–	2	–	–	–	–	–	–	–
WL711 and HD2329	PH132 and PH133	100	RILs/NILS	78	–	–	–	–	–	–	–	–	—	–	–	–	13
W7984	Prinz	72	BC2F3	210	11	8	8	–	–	–	–	–	–	–	–	–	–
Renan	Récital	194	RILs	212	1	5	–	–	–	–	–	–	–	7	3	2	11
Avalon	Habbit	200	RILs	60	–	—	–	–	–	–	–	–	–	–	–	–	7
Flair	XX86	111	BC2F3	197	9	14	2	8	–	–	–	5	–	–	–	–	–
RL4452	AC Domain	182	DH	322	3	–	–	–	–	–	–	–	–	5	5	6	8
AC Karma	87E03-S2B1	414	DH	489	3	3	–	–	–	2	–	–	–	–	–	–	–
Karl 92	TA 4152-4	190	BC2F1	666	2	–	1	1	–	–	–	–	–	–	–	–	–
Arche	Récital	222	DH	200	11	–	–	–	–	–	–	–	1	16	–	5	21
Opata85	W7984	110	RILs	521	3	–	5	6	2	–	–	–	–	–	–	–	–
WL711	PH132	110	RILs	173	2	–	1	–	–	–	–	–	–	–	–	–	–
Chuan 35050	Shannong 483	131	RILs	404	–	2	–	1	–	–	–	–	–	–	–	–	–
Superb	BW278	402	DH	268	1	1	–	–	3	–	–	–	–	–	–	–	–
TA4152-60	ND495	120	DH	746	–	–	–	–	–	2	2	–	–	–	–	–	–
Chuan 35050	Shannong 483	131	RILs	381	–	4	–	1	–	–	–	–	–	–	–	–	–
Heshangmai9	Yu8679	142	RILs	1,142	–	4	–	–	–	2	–	–	–	–	–	–	–
Halberd	Cutter	64	RILs	700	–	1	–	2	–	–	–	–	–	–	–	–	–
Chuanmai42	Chuannong16	127	F2	1,912	–	2	–	1	–	–	–	–	–	–	–	–	–
Laizhou953	Am3	166	RILs	857	–	–	–	1	–	–	1	–	–	–	–	–	–
Line3228	Jing 4839	237	F2	1,125	–	3	–	2	–	–	4	–	–	–	–	–	–
RAC875	Kukri	368	DH	850	1	2	–	–	–	–	–	–	–	–	–	–	–
Rye Selection111	Chinese Spring	230	RILs	836	–	2	–	–	–	–	–	–	–	–	–	–	–
Hanxuan10	Lumai 14	150	DH	395	1	–	–	–	–	–	–	–	–	–	–	–	–
Total					221								73				82

*Table columns deliver references for the populations used such as, name of parental lines (P1, P2), population size, type of population, number of lines, and QTL related to yield (T/h, Tons per Hectare; TKW, Thousand Kernel Weight; Tn, Tiller Number; GrN, Grain number; GrW, Grain Weight; HI, Harvest Index; PH, Plant Height; SL, Spike Length), grain protein content, and baking quality (Visco, Pentosan Viscosity; W, Dough Strength; Vol, Volume; and other BQ-related traits: Panif, Panification; Dur, Durity; L, Height of Bubble in Alveograph; Pel-Schank; Tst, Total Starch; Fsw, Flour Swelling Volume; Psi, Particule Size Index; Msap, Slope after peak of mixograph; Fsta, Farinograph test) and the total number of QTLs respectively. Table rows represent the 27 populations involved in the study*.

Three hundred and seventy-six QTLs (221 GY, 82 GPC, 73 BQ) projected on the consensus genetic map, were statistically combined through meta-analysis using Biomercator v3.0 to deliver MQTLs, defined as a locus where independent QTLs originating from at least two initial populations overlap and then computed to deliver consensus confidence intervals (CI). Following this strategy, the 376 projected QTLs produced 32 MQTLs including 18 for GY, 8 for GPC and 6 BQ (Figure 2 and Table 3). For GY, the 18 MQTLs involve 2 (on chromosomes 1B, 2B, 3B, 5A, 5B, 6A), 3 (on chromosomes 1D, 2A, 2D, 3B, 6B, 7A, 7D), 4 (on chromosomes 2D, 3D, 4B, 5A), and 5 (on chromosome 4A) yield components. 17 (94%) GY MQTLs involve tons per hectare (T/h) and thousand kernel weight (TKW) components among which 10 (56%) MQTLs also involve grain number (GrN). For BQ, the 73 QTLs produced 6 MQTLs involving at least one of the major traits of viscosity (Visco.), dough strength (W) and volume (Vol.). For GPC, eight MQTLs were identified involving chromosomes 1A, 2A, 2B, 2D, 3A, 4A, 6B, and 7A.

**Table 3:** Wheat metaQTLs and associated candidate genes.

**MQTL**			**CI**		**cM**	**QTL**	Trait^*^	**Pop**	**Marker**	**Gene**	**Candidate gene**
**#**	**Trait**	**Chr**	**Left**	**Right**							
1	GY	1B	73,44	81,62	8,18	5	Tn, TKW	4	1,138	641	OsUGE1
2	GY	1D	134,90	172,00	37,10	4	Th, TKW, GrN	3	707	403	Adh
											ATPase
3	GY	2A	132,85	148,56	15,71	5	Th, TKW, GrN	4	270	334	NA
4	GY	2B	82,27	95,76	13,49	6	Th, TKW	3	669	652	GIF1
											crp1
5	GY	2D	51,79	67,70	15,91	13	Th, TKW, GrN, HI	3	676	55	PpdD1
6	GY	2D	129,37	168,19	38,82	6	Th, TKW, GrN	4	1,777	772	Compact spike gene
											3 pistils per floret
											Prog1
7	GY	3B	58,72	86,04	27,32	6	Th, TKW, GrN	4	1,537	1,666	Brittle rachis 3
											ATPase
											Gn1-a
											NYC1
8	GY	3B	159,98	207,40	47,42	4	TKW, GrN	3	1,804	408	Phytoclock1, GARP protein
9	GY	3D	84,21	102,07	17,86	7	Th, TKW, GrN, SL	5	1,048	1,070	GoGat
											Lsk1
10	GY	4A	100,12	139,38	39,26	8	Th, TKW, GrN, Tn, SL	3	140	165	SRS5
11	GY	4B	82,87	89,88	7,02	12	Th, TKW, GrN, SL	7	939	608	Gibberellin response modulator
											emp4
12	GY	5A	42,68	74,25	31,57	4	Th, TKW	3	4,145	1,772	DEP1
13	GY	5A	158,70	182,55	23,85	6	Th, TKW, GrN, HI	4	300	190	C17648
14	GY	5B	188,35	214,46	26,12	5	Th, TKW	3	1,411	108	OsNaPRT1
15	GY	6A	79,93	108,72	28,80	5	Th, TKW	4	1,369	1,989	TOC I
											Cry2
											Gw2
											FUWA
											EP3
											GS2
16	GY	6B	100,39	106,93	6,54	3	Th, TKW, SL	3	114	46	NA
17	GY	7A	78,75	106,12	27,37	7	Th, TKW, GrN	4	400	162	incw2
											MOC1
											SSG6
18	GY	7D	92,09	97,63	5,54	7	Th, TKW, GrN	5	263	30	Rc3
19	GPC	1A	48,66	75,55	26,89	2	GPC	2	1,847	548	GliA3
											Tri
20	GPC	2A	93,52	106,85	13,33	3	GPC	2	1,084	306	RuBisCO
21	GPC	2B	68,01	73,84	5,83	5	GPC	3	488	131	NA
22	GPC	2D	103,52	117,20	13,69	6	GPC	2	268	65	NA
23	GPC	3A	65,43	71,33	5,90	8	GPC	2	470	476	Vivip-1
24	GPC	4A	72,15	95,71	23,56	3	GPC	2	240	37	NA
25	GPC	6B	88,17	94,87	6,70	4	GPC	3	472	30	NA
26	GPC	7A	156,71	171,82	15,11	3	GPC	2	164	17	NA
27	BQ	1A	75,55	88,69	13,15	5	W, Vol	3	3,294	2,334	GluA1
28	BQ	1B	84,81	99,54	14,74	7	Visco, Fsw, Msap	2	358	152	NA
29	BQ	1B	124,80	135,99	11,19	4	Visco, W	2	147	33	NA
30	BQ	3D	134,45	138,23	3,78	5	Visco, W, Panif	2	23	33	NA
31	BQ	4B	79,14	85,97	6,83	3	W, Fsta	2	540	230	NA
32	BQ	7A	194,75	204,32	9,57	4	Vol, Pelsc, Tst	3	528	309	NA

*The table delivers the detailed informations for the 32 MQTLs (rows) with, in columns, the trait (GY, GPC, or BQ), the chromosome, the confidence interval (left and right borders), the flanking markers (left and right borders), the genetic distance (in centiMorgans, cM), the number of QTLs involved, the trait components, the number of populations involved, the number of markers available, the number of genes from the syntenome and the list of candidate genes*.

* *cf trait nomenclature in Table 2 legend*.

The 32 MQTLs were located on all chromosomes except 4D, 5D, 6D, and 7B. The most precise MQTLs (i.e., reduced confidence intervals) were located on chromosomes 3D (3.78 cM) for BQ, 7D (5.54 cM) for GY, and 2B (5.83 cM) for GPC. Finally, from the initial set of 376 QTLs, 171 (45%) have been involved in the calculation of the final repertoire of 32 MQTLs and deriving from 24 (89%) of the 27 considered populations. The 32 MQTLs and associated QTLs are made available as Supplementary Tables 2–8.

### Synteny-based candidate genes of wheat traits

The consensus genetic map (140,315 markers) associated with QTLs (376) and derived MQTLs (32) were projected on the wheat syntenome consisting of 99,386 gene models covering 10.2 Mb of sequence fragmented into 10.8 million of scaffolds (Borrill et al., 2015), Figure 1. We recently produced the most accurate synteny-based gene order in wheat, referenced to as syntenome (Pont et al., 2011, 2013), where the most robust wheat genetic map involving 40,267 markers (Wang et al., 2014), and delivering 13,168 orthologous relationships with the ancestral grass genome (Murat et al., 2014), was enriched with 59,732 wheat syntenic (ancestral) genes/scaffolds intercalated between molecular markers, ultimately delivering 72,900 (73.4% of the 99,386 gene models) ordered genes on the 21 chromosomes (El Baidouri et al., 2017; Pont and Salse, 2017). Wang et al. (2014) genetic map was used as a backbone to project the consensus genetic map (14,0315 markers) and associated QTLs (376) and MQTLs (32) onto the wheat syntenome (72,900 genes) to deliver a robust list of candidates for each of the MQTL intervals. We then deliver an exhaustive list of 15,772 genes under the 32 MQTL CIs for further validation (Figure 2, Supplementary Tables 2–8). Among this repertoire of wheat genes, we identified 37 major candidates from known and validated genes in grass relatives (Sakamoto and Matsuoka, 2008; Huang et al., 2013; Valluru et al., 2014; Agarwal et al., 2016), Table 3. Regarding yield, the candidate genes can be classified into developmental genes (13 genes), genes linked to metabolism (11 genes), genes driving grain size (3 genes), and genes involved in grain number and grain weight (2 genes). Regarding grain protein content we identified *Gliadin, Triticin, Tri-ribulose-1,5-bisphosphate carboxylase/Viviparous*, as candidates for three MQTLs (on chromosomes 1A, 2A, 3A) with five additional MQTLs without any obvious candidates. Finally, for the six MQTLs of baking quality, only a *Glutenin* has been proposed as candidate for a single MQTL located on the chromosome 1A.

The current data are made accessible to the scientific community through a web platform allowing to navigate between the genetic and genomic resources, from QTL, MQTL up to the synteny with grass relatives and ultimately candidate genes. The public web interface named PlantSyntenyViewer available at http://urgi.versailles.inra.fr/synteny-wheat (Figure 2) delivers (either through a chromosome or gene search), the genetic (markers, QTLs, MQTLs) and associated genomic (wheat syntenome and syntenic genes from related grasses) data that can be considered for (i) marker development, (ii) improving conserved gene annotation or (iii) candidate gene selection for any traits of interest (either GY, GPC, BQ, or trait components). Such resources can also be considered for a translational research approach with grass relatives where the delivered QTL, MQTL, and candidate genes in wheat are projected on rice, and sorghum genomes and can then be also considered as candidates of major traits for such closely related species.

## Discussion

The access to 27 public quantitative genetic studies from the last decades offered the opportunity to unveil major loci driving agronomical traits in wheat. Meta-analysis of QTLs can be considered as a statistical tool that helps in combining data from different sources into a single study through the identification of the relevant subset of genome loci (MQTL) which are dominant in different genetic populations for the considered traits. This strategy of meta-analysis shows that the MQTL generally gives a confidence interval that is confirmed through numerous single independent studies. Moreover, the identification of a narrow genetic/genomic confidence interval driving traits, delivered by the inferred MQTLs, is a key step for a more precise search for relevant candidate genes (Veyrieras et al., 2007). Based on the construction of a consensus genetic map with 140,315 molecular markers, we integrated 376 QTLs into 32 MQTLs consisting in 18, 8, 6 MQTLs for, respectively, yield, baking quality and grain protein content. Such high resolution and large-scale integration of wheat genetic and genomic resources offers a tremendous set of gene-based markers that can be considered as a guide for accelerated dissection of major agronomical traits in breeding.

Jordan et al. (2007) used the RL4452 × “AC Domain” population (included in our data set) to map expression level polymorphisms and identified 542 eQTLs considered as representing major effectors of yield, baking quality, and grain protein content. This approach is complementary to our data as it can provide regulatory candidates of our inferred 32 MQTLs. Moreover, there were a few regions of the genome in which eQTL clustered (hot spots) that may represent chromosomal regions affecting the expression of several genes. These results are consistent with the current data set as the eQTL clusters reported in this study do correspond to MQTLs involved in multiple traits in our analysis. As example, a 4B loci (between *BF484674-297* and *WMC349*) associated with 20 eQTL from Jordan et al. (2007) corresponds to MQTL11 for yield (TKW, Th, GrN), and MQTL31 for baking quality (Fst, W) in the current study (Table 3).

The 376 QTLs and derived 32 MQTLs were projected onto the wheat syntenome consisting in 72,900 ordered genes on the 21 chromosomes delivering direct links between wheat and rice-*Brachypodium*-sorghum genomes. We delivered 15,772 genes covering the 32 MQTLs intervals, including 37 major candidates based on known genes in wheat and grass relatives (Valluru et al., 2014). Our study consists in a clear example illustrating the power of the translational research approach in exploiting the knowledge gained in relatives (rice, sorghum, and *Brachypodium* here) to dissect the genetic basis of major traits in a more complex species (wheat in our case). These candidate genes are best guesses, according to their function and mapping position in relation to the MQTL confidence intervals. However the co-location criteria between MQTLs and genes do not assume or even prove any functional relationship. The 37 synteny-based genes proposed for the 32 MQTLs can be considered as potential candidates for future functional validation. These genes located under MQTL intervals have to be only considered as best candidates for cloning, functional analyses or the development of markers for crop improvement (i.e., marker assisted breeding programs). Major wheat genes of yield components have already been identified in the past through similar approaches taking advantages of related species such as GW2 (Bednarek et al., 2012), GS3 (Li et al., 2016), CKX2 (Zhang et al., 2011), ISA3 (Kang et al., 2013), Eps (Faricelli et al., 2010), IPA1 (Li et al., 2016), DEP1 (Li et al., 2016).

Overall, the current study of meta-analysis of QTL in wheat clearly shows that MQTLs (32) are associated with confidence interval that are confirmed through numerous single independent analyses and associated with relevant genes (15,772 including 37 major candidates) and derived markers (28,630 SNP-based makers) to be considered in current breeding schemes. These resources are now made available through the web interface PlantSyntenyViewer for cloning, functional analyses, or the development of markers for wheat improvement.

## Materials and methods

### Construction of the consensus genetic map

Four public wheat genetic maps were considered for the construction of a wheat consensus genetic map: Xu et al. (2008) with 2,293 markers, Somers et al. (2004) with 1,239 markers, Wang et al. (2014) with 40,267 markers and Saintenac et al. (2013) with 104,804 markers. Biomercator v3.0 (Sosnowski et al., 2012) delivers a graphical interface that allows the projection of different maps into a single genetic consensus map. A text file is necessary to describe all the genetic maps (marker name, position) as well as their associated QTL statistics (LOD score, R^2^ percentage of phenotypic variation, confidence interval). Biomercator first integrates independent genetic maps into a comprehensive map (with a specific map projection algorithm) and secondly recalculates the marker position as well as those of the initial QTLs, based on a most likely consensus QTL distribution through meta-analysis algorithms. As a consequence, we used the first function of Biomercator v3.0 to compile four genetic maps to create a dense consensus genetic map with all the markers available from the investigated maps. A prerequisite for producing a comprehensive consensus genetic map is to eliminate inconsistent markers, i.e., markers located on non-identical positions between two maps, so that they could not create discrepancies in the final consensus map. As a consequence, we used the MapInspect software (http://mapinspect.software.informer.com/), to verify chromosome by chromosome the marker order between the four considered maps. All the inconsistent loci (mainly non-collinear markers corresponding to large inversions) were thus discarded. Following this strategy we deliver a wheat consensus genetic map consisting of 140,315 markers (Table 1 and Supplementary Table 1).

### QTL projection and MQTL construction

Twenty seven genetic maps from independent quantitative genetic studies (Charmet et al., 2001; Börner et al., 2002; Groos et al., 2003, 2007; Prasad et al., 2003; Huang et al., 2004, 2006; Turner et al., 2004; Kulwal et al., 2005; McCartney et al., 2005, 2006; Kumar et al., 2006; Narasimhamoorthy et al., 2006; Laperche et al., 2007; Li et al., 2007; Chu et al., 2008; Cuthbert et al., 2008; Sun et al., 2008; Wang et al., 2009, 2011; Deng et al., 2011; Tang et al., 2011; Bennett et al., 2012; Mir et al., 2012; Ravel et al., 2012; Liu et al., 2013) of grain yield (GY with grain number, tons per hectare, tiller number, thousand kernel weight, harvest index, plant height), GPC, and baking quality (BQ with pentosan viscosity, dough strength, volume, panification, durity, height of bubble in alveograph, pel-schank, total starch, flour swelling volume, particule size index, slope after peak of mixograph, farinograph test), as well as 110 public morpho-physiological genes (height, protein content, gluten subunits, gliadins, pigmentation, vernalization, glaucousness, tiller inhibition, brittle rachis, etc.) were projected on the consensus genetic map using common markers available in the Komugi gene list (McIntosh et al., 2011) and the constructed consensus genetic map.

The 27 genetic maps with QTL/loci mapped were projected on the consensus genetic map by means of the homothetic function of Biomercator v3.0. All the ambiguous loci being previously detected by MapInspect were removed as described above. Biomercator then detects all the markers in each single genetic map (source of QTLs) and locates them onto the consensus map on the basis of their relative distance compared to common markers, and then projects the most likely position of each QTL with their left and right flanking borders of confidence intervals (using an homothetic function based on common markers between the reference map and the QTL map). All the projections have to be cross-verified because small inversions would not allow a precise projection of QTLs (based on the positions of QTL and markers associated with confidence intervals), Table 2.

After integration of the 27 genetic maps (and associated 376 QTLs) into the consensus genetic map using Biomercator v3.0, meta-analysis of QTLs (as described by Goffinet and Gerber, 2000) was launched for each trait separately. The approach provides decisions, based on a modified Akaike criterion, to determine the number of MQTL that best fits the QTLs available on different genetic maps. Biomercator v3.0 determines if n QTL detected from independent experiments in the same region of a chromosome are consistent with 1, 2, 3, 4, or n MQTL models (the n MQTL model being the case where there are as many MQTLs as input QTLs). For each of these five models, Biomercator predicts the most likely QTL distribution using means of the maximum likelihood method (ratio likelihood method). Then, an Akaike-type statistical criterion indicates the best model among the five available. Models with least Akaike values predicted on each chromosome was used to identify MQTLs on the chromosomes. Currently, the method used in the software does not allow to distinguish between models with more than four MQTLs on the same linkage group. If the estimated number of MQTLs is more than four, Biomercator declares that the most probable model is a number of MQTLs equal to the number of initially projected QTLs (n MQTL model). In this case, the deletion function of the software was used to select segments of a linkage group separated by regions with no QTL and the meta-analysis was applied to these segments. Following this strategy, we identified 32 MQTLs for respectively yield (18), baking quality (6), and grain protein content (8) traits (Table 3 and Supplementary Tables 2–8).

### Genetic map and associated MQTL projection of the wheat syntenome

The wheat syntenome was used as described in El Baidouri et al. (2017). Briefly, the ancestral grass genome (AGK for ancestral grass karyotype) was used as delivered in Murat et al. (2014) with 58,933 ordered ancestral genes on 12 ancestral chromosomes based on synteny relationships investigated between *Oryza sativa* (International Rice Genome Sequencing Project, 2005), *Brachypodium distachyon* (International Brachypodium Initiative, 2010) and *Sorghum bicolor* (Paterson et al., 2009) genomes. The blastn alignment of 40,267 mapped markers from the wheat consensus SNP map published by Wang et al. (2014) and AGK genes, delivered orthologs between these two resources. Using DRIMM-Synteny tool (Pham and Pevzner, 2010), we built synteny groups allowing the identification of ancestral regions as well as the ancestral gene content and order between wheat markers along the consensus map (21 chromosomes). Following this method, we ordered 62,135 wheat sequence scaffolds (from International Wheat Genome Sequencing Consortium, 2014) containing 72,900 genes along the 21 chromosomes of the bread wheat genome, i.e. referenced as the bread wheat syntenome (available at http://urgi.versailles.inra.fr/synteny-wheat). The genetic map from Wang et al. (2014), used as a backbone for both the consensus genetic map and the syntenome, provided direct links between both resources to project the 376 QTLs and associated 32 MQTLs, ultimately delivering a complete repertoire of genes (from the syntenome) covering the MQTL intervals (from the consensus genetic map) in wheat, rice, sorghum, and *Brachypodium* (Table 3).

### Synteny-based identification of candidate genes

A list of 110 genes located on the four genetic maps used in this study as well as candidate genes available in grass relatives (mainly rice) from Sakamoto and Matsuoka (2008), Huang et al. (2013), Valluru et al. (2014), and Agarwal et al. (2016) were considered. The genes were aligned (blastn) against the wheat syntenome using two parameters to increase the stringency and significance of the sequence alignment: cumulative identity percentage (CIP) corresponding to the cumulative percent of sequence identity obtained for all the high scoring pairs (HSPs) and CALP for the cumulative alignment length percentage (Salse et al., 2009). The CIP and CALP parameters allow the identification of the best alignment, i.e., the highest cumulative percentage of identity in the longest cumulative length, taking into account all HSPs obtained for any pairwise alignment. Following this strategy, 37 synteny-based candidates were proposed for the 32 MQTLs (Table 3 and Supplementary Table 9). The integrated genomic and genetic resources are made available to the scientific community through a user friendly and public web interface PlantSyntenyViewer available at http://urgi.versailles.inra.fr/synteny-wheat delivering QTLs (376), MQTLs (32), genes (15,772), major candidates (37), and derived SNP markers (28,630).

## Author contributions

UQ, CP, and QA performed the analysis. RF, LB, MA, and HQ managed the web viewer PlantSyntenyViewer. UQ and JS wrote the article.

### Conflict of interest statement

The authors declare that the research was conducted in the absence of any commercial or financial relationships that could be construed as a potential conflict of interest.

## Supplementary material

Supplementary Table 1 Wheat consensus genetic map.

Supplementary Table 2 Wheat chromosome group 1 (A, B, and D subgenomes) with genetic maps, QTLs, MQTLs, and candidate genes.

Supplementary Table 3 Wheat chromosome group 2 (A, B, and D subgenomes) with genetic maps, QTLs, MQTLs, and candidate genes.

Supplementary Table 4 Wheat chromosome group 3 (A, B, and D subgenomes) with genetic maps, QTLs, MQTLs, and candidate genes.

Supplementary Table 5 Wheat chromosome group 4 (A, B, and D subgenomes) with genetic maps, QTLs, MQTLs, and candidate genes.

Supplementary Table 6 Wheat chromosome group 5 (A, B, and D subgenomes) with genetic maps, QTLs, MQTLs, and candidate genes.

Supplementary Table 7 Wheat chromosome group 6 (A, B, and D subgenomes) with genetic maps, QTLs, MQTLs, and candidate genes.

Supplementary Table 8 Wheat chromosome group 7 (A, B, and D subgenomes) with genetic maps, QTLs, MQTLs, and candidate genes.

Supplementary Table 9 Detailed information regarding the 37 candidate genes.
